# Availability of web servers significantly boosts citations rates of bioinformatics methods for protein function and disorder prediction

**DOI:** 10.1093/bioadv/vbad184

**Published:** 2023-12-25

**Authors:** Jiangning Song, Lukasz Kurgan

**Affiliations:** Biomedicine Discovery Institute and Department of Biochemistry and Molecular Biology, Monash University, Clayton, VIC 3800, Australia; Monash Data Futures Institute, Monash University, Clayton, VIC 3800, Australia; Department of Computer Science, Virginia Commonwealth University, Richmond, VA 23284, United States

## Abstract

**Motivation:**

Development of bioinformatics methods is a long, complex and resource-hungry process. Hundreds of these tools were released. While some methods are highly cited and used, many suffer relatively low citation rates. We empirically analyze a large collection of recently released methods in three diverse protein function and disorder prediction areas to identify key factors that contribute to increased citations.

**Results:**

We show that provision of a working web server significantly boosts citation rates. On average, methods with working web servers generate three times as many citations compared to tools that are available as only source code, have no code and no server, or are no longer available. This observation holds consistently across different research areas and publication years. We also find that differences in predictive performance are unlikely to impact citation rates. Overall, our empirical results suggest that a relatively low-cost investment into the provision and long-term support of web servers would substantially increase the impact of bioinformatics tools.

## 1 Introduction

Scientific articles that concern bioinformatics methods and databases are substantially over-represented among the most-cited scientific literature (Wren 2016, Wren *et al.* 2017). A study has shown that about one-third of the most cited papers in science were bioinformatics software/methods papers, which corresponds to 31-fold enrichment relative to the total number of these articles (Wren 2016). However, while some of these resources are very widely cited and used, many garner much less attention. One factor associated with high levels of citations is the continued availability of these resources (Wren *et al.* 2017). Moreover, several recent studies of broader collections of scientific papers identified other factors that affect citation rates, such as proportion of jargon words in the title and abstract (Martinez and Mammola 2021), quality of writing (clarity, creativity, and narrative structure) (Ryba *et al.* 2019), and certain characteristics of their reference lists (Mammola *et al.* 2021).

Here, we focus on the bioinformatics methods and investigate whether their availability and how they are provided to the end users have impact on their citation rates. There are hundreds of these methods (Zhang *et al.* 2011, Miao and Westhof 2015, Yan *et al.* 2016, Jiang *et al.* 2017, Katuwawala *et al.* 2019a,b, Liu *et al.* 2019, Necci *et al.* 2021, Zhao and Kurgan 2021, Zhang *et al.* 2022, Basu *et al.* 2023). They are available as web servers that can be used remotely without the need to install and to use local computing resources and/or standalone code that has to be installed and run locally by the users, which typically requires more computational expertise. Maintaining a web server depends usually on continued employment at the same institution and, sometimes, continued funding when a non-trivial amount of maintenance is needed. Code also has to be maintained to be compatible with newer versions of operating systems and third-party software that are needed to run it. These two options offer complementary benefits where web servers are typically easier to use but are limited in the size of the inputs they can process, while standalone code can be run on a larger scale and is easier for embedding into other bioinformatics applications. Moreover, in some cases neither code nor web server are provided, which means that these methods are only described by the authors and have to be reimplemented by the users. We empirically study the relation between the mode of availability and the corresponding citations rates for a large collection of 100 recently published protein bioinformatics methods. We also investigate potential impact of differences in the predictive performance on the citations.

## 2 Methods

We select a diverse collection of protein bioinformatics methods from three distinct areas: (i) Functions of structured proteins, which cover prediction of protein–protein, protein–DNA, protein–RNA, and protein–peptide interactions (Zhang *et al.* 2011, Miao and Westhof 2015, Yan *et al.* 2016, Jiang *et al.* 2017, Zhang *et al.* 2022); (ii) Functions of intrinsically disordered proteins and protein regions that include prediction of disordered linkers and protein–protein, protein–DNA, protein–RNA, protein–peptide, and protein–lipid interactions (Katuwawala *et al.* 2019a,b, Basu *et al.* 2023); and (iii) Intrinsic disorder (Liu *et al.* 2019, Necci *et al.* 2021, Zhao and Kurgan 2021, Kurgan *et al.* 2023). We cover the three research areas to investigate whether our conclusions/observations are consistent across different communities that develop predictors. We did not include prediction of structure since this area is now dominated by AlphaFold2 (Jumper *et al.* 2021a,b, Tunyasuvunakool *et al.* 2021, Varadi *et al.* 2022), and we expect that only a few new methods in the area will be released in the near future. In contrast, AlphaFold2 is outperformed by other tools in the context of the prediction of intrinsic disorder (Wilson *et al.* 2022, Zhao *et al.* 2023). For instance, using the DisProt benchmark dataset from a recent Critical Assessment of protein Intrinsic Disorder prediction (CAID) experiment (Necci *et al.* 2021), AlphaFold2 predicts disorder with the area under receiver operating characteristic curve (AUC) and area under the precision–recall curve (AUPRC) of 0.785 and 0.357, respectively, compared to the best disorder predictor on this dataset, flDPnn (Hu *et al.* 2021), which obtains AUC of 0.814 and AUPRC of 0.475 (Zhao *et al.* 2023).

We select tools that were published in the last 10 years (2011–2021 inclusive), excluding methods published in 2022 or newer since such tools are too new to accumulate reliable citation data. We settle for the 10-year window to include relatively recent methods, be able to analyze results longitudinally, and reduce the impact of difficulty/inability with deploying web server for the older methods. We use a combination of PUBMED searches, surveys (Zhang *et al.* 2011, Miao and Westhof 2015, Si *et al.* 2015a,b, Varadi *et al.* 2015, Yan *et al.* 2016, Dosztányi and Tompa 2017, Jiang *et al.* 2017, Meng *et al.* 2017a,b, Katuwawala *et al.* 2019a,b, Liu *et al.* 2019, Barik and Kurgan 2020, Katuwawala and Kurgan 2020, Necci *et al.* 2021, Zhao and Kurgan 2021, Cui *et al.* 2022, Kurgan 2022, Zhang *et al.* 2022, Basu *et al.* 2023, Uversky and Kurgan 2023, Zhao and Kurgan 2023), and Google scholar citations scanning to identify a comprehensive collection of methods. We list these methods in Supplementary Table S1 (43 predictors of functions of structured proteins), Supplementary Table S2 (24 predictors of functions of intrinsically disordered proteins), and Supplementary Table S3 (33 predictors of intrinsic disorder). The tables also record citations for these methods that we collected from Google Scholar in May 2023. We primarily rely on the annual citation rate that is defined as the total number of citations divided by the number of years since publication.

**Figure 1.**
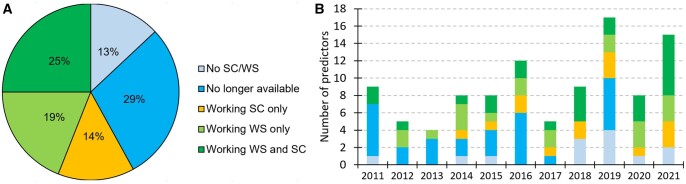
Availability of bioinformatics methods for the prediction of protein function and disorder; SC (standalone code); WS (web server); no SC/WS (no standalone code and web server; description only); methods denoted as “No longer available” were available at the time when they were published but they were inaccessible as of May 2023 when we attempted to access them. Panel (A) shows the overall breakdown. Panel (B) shows how these data distribute over the publication/release time.

Figure 1A reveals that the availability of these methods covers the entire spectrum of options rather uniformly. On the two extreme ends, 25% of methods are available as both standalone code (SC) and web server (WS) while 13% have no SC and no WS (“no SC/WS”). Moreover, 29% of methods initially were made available while later (i.e. in May 2023 when we checked twice, about two weeks apart) their SC and WS could not be accessed, rendering them unavailable. Among these “no longer available” methods, 21%, 65%, and 14% were originally provided as SC, WS and both, respectively. Altogether, 42% of methods have no SC/WS or are no longer available, compared to 44% that have WS and 39% that have SC. Figure 1B summarizes the five groups of methods (no SC/WB; no longer available; working SC only; working WS only; working WS and SC) over time when they were published. While in general we observe a mixture of different modes of availability/unavailability over time, the last couple of years have a larger proportion of the working web servers (the two shades of green combined). More precisely, nearly 70% of the methods published in 2022 and 2021 have working web servers, whereas this rate goes down to between 31 and 50% in the preceding 2-year windows. However, some of these recently released web servers will inevitably stop being supported (i.e. join the “no longer available” group), which is apparent when looking at the rate of the “no longer available” (blue) tools in the earlier years.

## 3 Results

### 3.1 Methods with working web servers are significantly more highly cited

**Figure 2.**
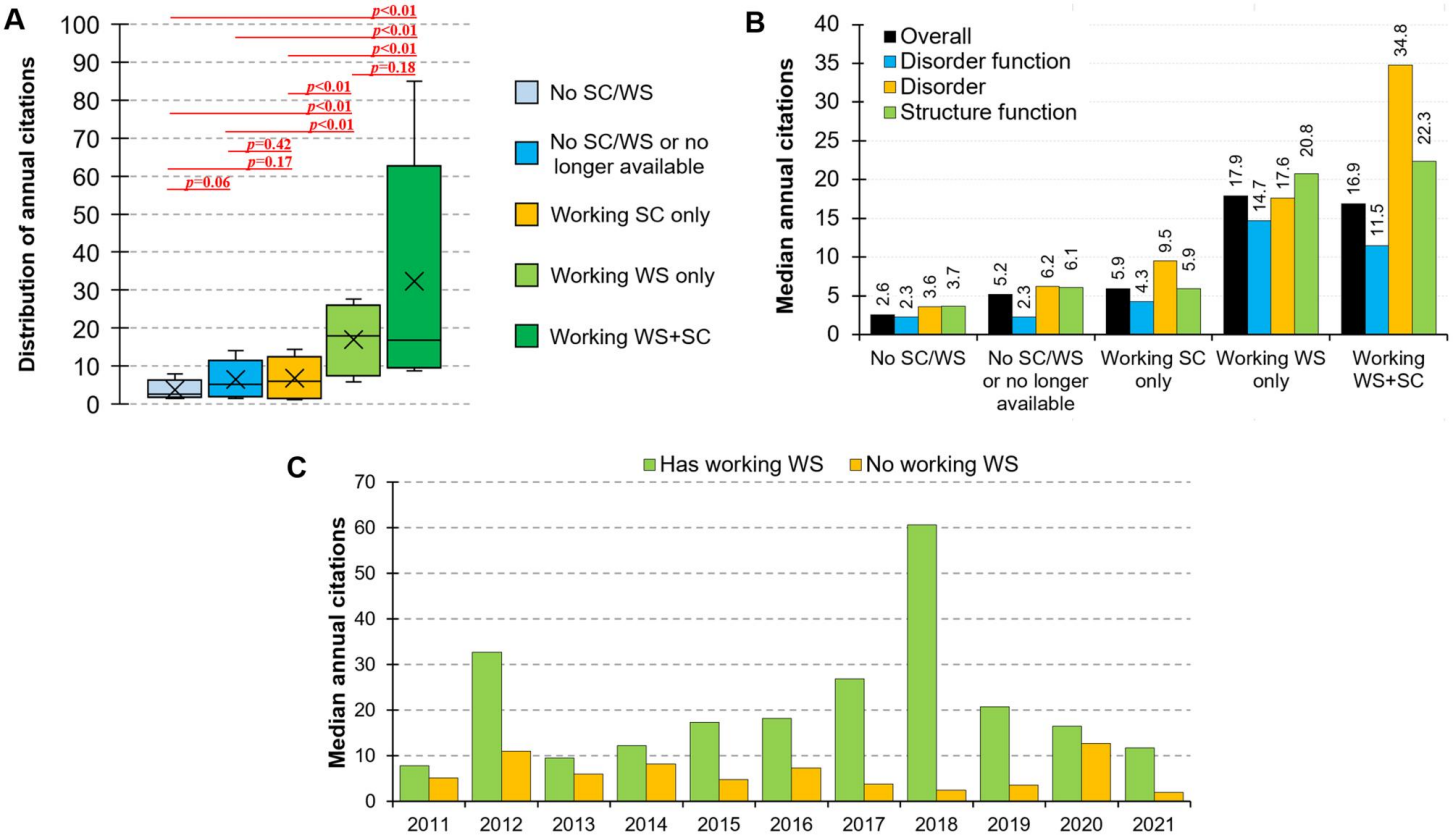
Analysis of the annual citation rates. (A) Distribution of the annual citation rates for the entire set of 100 methods; SC (standalone code); WS (web server). Box plots show the 1st quartile, median (line in the middle), and third quartile, while whiskers are first and nineth deciles; “X” denotes the average. We computed the *P*-values using the Wilcoxon-Mann-Whitney test (Marx *et al.* 2016). (B) Comparison of median citation rates across different prediction areas. (C) Comparison of median citation rates analyzed by the year of publication.

Our central question is whether the type of availability is associated with differences in the citation rates. Figure 2A shows distributions of the annual citation rates and *P*-values that quantify statistical significance of differences in these rates for the five groups of methods: available as working standalone code (SC) and web server (WS); available as working WS; available as working SC; no SC/WS (at the time of publication); and no SC/WS or no longer available. We find that the annual citations are rather low for methods with no SC/WS (median citations = 2.6), no SC/WS or no longer available (median = 5.2) and available as working SC (median = 5.9), and the differences between these three collections of methods are not statistically significant. However, methods that have working WS and that have working WS and SC have significantly higher citations rates compared with the above three groups (median = 17.9 and 16.9, respectively; *P*-value < .01), while the differences between these two groups are not significant. Furthermore, we investigate whether the inclusion of highly cited (“outlier”) articles could skew the results. To do that, we compute the medians and perform statistical tests when excluding the top 10% of the most cited articles in each group of methods. The corresponding medians are 2.5 for the no SC/WS, 4.6 for the no SC/WS or no longer available, 5.9 for the working SC, 17.4 for the working WS, and 15.7 for the working WS and SC groups of methods. Similarly, the *P*-values when comparing these groups of tools agree with the analysis that considers all methods, i.e. the only significant differences are between the first three groups (no SC/WS, no longer available, only working SC) and the latter two groups (working WS only, working WS and SC), *P*-values < .01; all other *P*-values are above .05. Overall, we find that the two sets of results are consistent, suggesting that the inclusion of the web servers is the main driver of substantially higher citation rates.

We also investigate robustness of this finding across the three different prediction areas (Fig. 2B) and over time (Fig. 2C). Overall, the disorder predictors enjoy a modestly higher annual citation rate (median = 14.5) compared to the other two area, disorder function prediction (median = 9.3) and structure function prediction (median = 8.0). In spite of these differences, we find that the increases in citation rate due to the inclusion of the web servers are consistent across the three prediction areas. The overall higher annual citation rate and the spike for the disorder predictors that have both working web servers and working code are primarily driven by a few highly cited tools, such as IUPred2A, DISOPRED3, ESpritz, and IUPed3. Moreover, Fig. 2C shows that the methods that have working web servers are more highly cited irrespective of when they were published.

Altogether, we find that bioinformatics methods that have working web servers are on average cited three times more often when compared to the methods that lack this feature. This increase is consistent across different prediction areas and time, suggesting that our finding is robust.

### 3.2 Citation rates are not determined by predictive performance

We examine whether predictive performance could be a confounding factor, i.e. whether methods that offer working web servers are also more accurate when compared with the tools that do not. We investigate this aspect for the disorder predictors using recently released results from a large community-driven evaluation, CAID (Critical Assessment of protein Intrinsic Disorder prediction) (Lang and Babu 2021, Necci *et al.* 2021, Zhao and Kurgan 2022). CAID results were produced by independent assessors, which exclude authors of the predictors, for a larger collection of methods using a sizeable benchmark dataset and well-established evaluation protocols. We note that subsequent analysis of the CAID results demonstrates that quality of the disorder predictors has improved over time, where newer methods provide more accurate predictions when compared to older tools (Zhao and Kurgan 2021). The other two areas lack such robust assessments and the results there are fragmented, where authors of individual tools compare typically small collections of predictors using different datasets.

**Figure 3.**
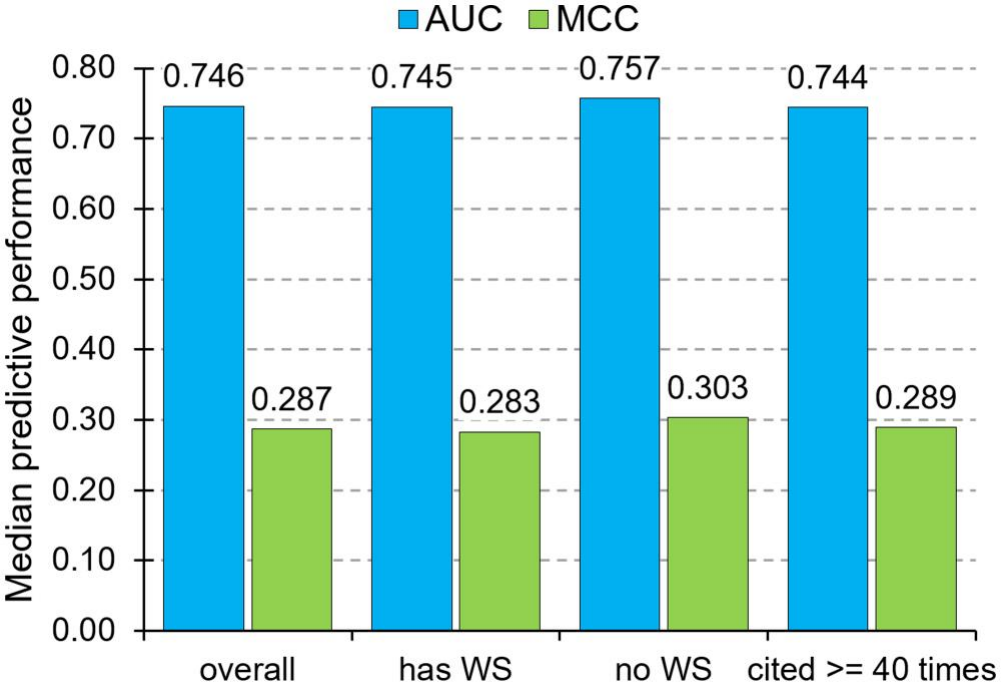
Comparison of median predictive performance for the intrinsic disorder prediction.

We found the CAID results for 15 of the 33 considered here disorder predictors (Supplementary Table S4). Figure 3 summarizes these results by comparing the median predictive performance quantified with two commonly used metrics, AUC (Area Under the ROC Curve) and MCC (Matthews Correlation Coefficient) that evaluate predicted propensities and binary predictions, respectively. We compare the overall predictive quality with the quality of the methods that have working web servers, that do not have web servers and that are highly cited (i.e. annual citation rates ≥ 40). We found that median predictive quality is nearly identical for these four collections of predictors. In fact, the highly cited methods secure a slightly lower performance, with the two highest cited tools (IUPred2A and DISOPRED3) obtaining below average results (AUC = 0.741 and 0.701, and MCC = 0.278 and 0.241, respectively). Moreover, the Pearson correlation coefficients between the annual citation rates and the AUC and MCC are −0.12 and −0.15, respectively. This suggests that higher levels of accuracy do not determine higher rates of citations, confirming that the availability of working web servers is the key driver of high citations.

## 4 Summary and discussion

While bioinformatics resources dominate lists of the most cited articles (Wren 2016), many of bioinformatics methods have relatively low citation rates. One of the key factors that influences citation rates is the continued availability of these resources (Wren *et al.* 2017). Our results support that finding based on the relatively low citation rates that we measured for the methods that are no longer available (median = 5.2) versus those that have working web servers (median = 17.9) (see Fig. 1A). More importantly, we find that bioinformatics methods that have working web servers generate on average three times as many citations compared to the methods that are available as only source code, that have no web server and no source code, or are no longer available. This is a robust observation that holds across different prediction areas and publication years, and which is not influenced by the differences in the predictive performance.

The development of bioinformatics methods is a long, complex and resource-hungry process that encompasses conceptualization, design, implementation, testing, deployment, and maintenance. The provision and long-term support of web servers is a relatively low-cost aspect of this process that seems to significantly improve citation rates, and by proxy also likely leading to increased rates of their use. We hope that our empirical findings will motivate more authors to build and maintain working web servers for their bioinformatics methods. We also believe that requiring the commitment to support web servers for an extended period of time at publication would substantially increase impact of bioinformatics tools, benefitting both the developers and users. The currently expected tool-support time ranges from 2 years for the application notes in the *Bioinformatics* journal, 3 years for the “Computation Resources for Molecular Biology” issue in *Journal of Molecular Biology*, and 5 years for the web server issue of the *Nucleic Acids Research* journal. Moreover, methods without web servers are still being published at a relatively high rate.

## Supplementary Material

vbad184_Supplementary_Data

## Data Availability

The data underlying this article are available in the article and in its online supplementary material.

## References

[vbad184-B1] Barik A , Kurgan LA. Comprehensive overview of sequence-based protein-binding residue predictions for structured and disordered regions. In: Gromiha M (ed.), Protein Interactions. Singapore: World Scientific Publishing, 2020, 33–58.

[vbad184-B2] Basu S , Kihara D, Kurgan L. Computational prediction of disordered binding regions. Comput Struct Biotechnol J 2023;21:1487–97. 36851914 10.1016/j.csbj.2023.02.018 PMC9957716

[vbad184-B3] Cui F , Zhang Z, Cao C et al Protein–DNA/RNA interactions: machine intelligence tools and approaches in the era of artificial intelligence and big data. Proteomics 2022;22:e2100197. 35112474 10.1002/pmic.202100197

[vbad184-B4] Dosztányi Z , Tompa P. Bioinformatics approaches to the structure and function of intrinsically disordered proteins. In: Rigden D (ed.), From Protein Structure to Function with Bioinformatics. Dordrecht: Springer Netherlands, 2017, 167–203.

[vbad184-B5] Hu G , Katuwawala A, Wang K et al flDPnn: accurate intrinsic disorder prediction with putative propensities of disorder functions. Nat Commun 2021;12:4438. 34290238 10.1038/s41467-021-24773-7 PMC8295265

[vbad184-B6] Jiang Q , Jin X, Lee S-J et al Protein secondary structure prediction: a survey of the state of the art. J Mol Graph Model 2017;76:379–402. 28763690 10.1016/j.jmgm.2017.07.015

[vbad184-B7] Jumper J , Evans R, Pritzel A et al Applying and improving AlphaFold at CASP14. Proteins2021a;89:1711–21. 34599769 10.1002/prot.26257 PMC9299164

[vbad184-B8] Jumper J , Evans R, Pritzel A et al Highly accurate protein structure prediction with AlphaFold. Nature2021b;596:583–9. 34265844 10.1038/s41586-021-03819-2 PMC8371605

[vbad184-B9] Katuwawala A , Ghadermarzi S, Kurgan L. Computational prediction of functions of intrinsically disordered regions. Prog Mol Biol Transl Sci2019a;166:341–69. 31521235 10.1016/bs.pmbts.2019.04.006

[vbad184-B10] Katuwawala A , Peng Z, Yang J et al Computational prediction of MoRFs, short disorder-to-order transitioning protein binding regions. Comput Struct Biotechnol J2019b;17:454–62. 31007871 10.1016/j.csbj.2019.03.013 PMC6453775

[vbad184-B11] Katuwawala A , Kurgan L. Comparative assessment of intrinsic disorder predictions with a focus on protein and nucleic acid-binding proteins. Biomolecules 2020;10:1636. 33291838 10.3390/biom10121636 PMC7762010

[vbad184-B12] Kurgan L. Resources for computational prediction of intrinsic disorder in proteins. Methods 2022;204:132–41. 35367597 10.1016/j.ymeth.2022.03.018

[vbad184-B13] Kurgan L , Hu G, Wang K et al Tutorial: a guide for the selection of fast and accurate computational tools for the prediction of intrinsic disorder in proteins. Nat Protoc 2023;18:3157–72. 37740110 10.1038/s41596-023-00876-x

[vbad184-B14] Lang B , Babu MM. A community effort to bring structure to disorder. Nat Methods 2021;18:454–5. 33875888 10.1038/s41592-021-01123-5

[vbad184-B15] Liu Y , Wang X, Liu B. A comprehensive review and comparison of existing computational methods for intrinsically disordered protein and region prediction. Brief Bioinform 2019;20:330–46. 30657889 10.1093/bib/bbx126

[vbad184-B16] Mammola S , Fontaneto D, Martínez A et al Impact of the reference list features on the number of citations. Scientometrics 2021;126:785–99.

[vbad184-B17] Martinez A , Mammola S. Specialized terminology reduces the number of citations of scientific papers. Proc R Soc Proc Biol Sci 2021;288:20202581. 10.1098/rspb.2020.2581 PMC8059506 33823673

[vbad184-B18] Marx A , Backes C, Meese E et al EDISON-WMW: exact dynamic programing solution of the Wilcoxon-Mann-Whitney test. Genomics Proteomics Bioinf 2016;14:55–61. 10.1016/j.gpb.2015.11.004 PMC4792850 26829645

[vbad184-B19] Meng F , Uversky V, Kurgan L. Computational prediction of intrinsic disorder in proteins. Curr Protoc Protein Sci2017a;88:2.16.1–12.16.14. 10.1002/cpps.28 28369666

[vbad184-B20] Meng F , Uversky VN, Kurgan L. Comprehensive review of methods for prediction of intrinsic disorder and its molecular functions. Cell Mol Life Sci2017b;74:3069–90. 28589442 10.1007/s00018-017-2555-4 PMC11107660

[vbad184-B21] Miao Z , Westhof E. A large-scale assessment of nucleic acids binding site prediction programs. PLoS Comput Biol 2015;11:e1004639. 26681179 10.1371/journal.pcbi.1004639 PMC4683125

[vbad184-B22] Necci M , Piovesan D, Tosatto SCE et al; DisProt Curators. Critical assessment of protein intrinsic disorder prediction. Nat Methods 2021;18:472–81. 33875885 10.1038/s41592-021-01117-3 PMC8105172

[vbad184-B23] Ryba R , Doubleday ZA, Connell SD. How can we boost the impact of publications? Try better writing. Proc Natl Acad Sci USA 2019;116:341–3. 30622212 10.1073/pnas.1819937116 PMC6329950

[vbad184-B24] Si J , Cui J, Cheng J et al Computational prediction of RNA-binding proteins and binding sites. Int J Mol Sci2015a;16:26303–17. 26540053 10.3390/ijms161125952 PMC4661811

[vbad184-B25] Si J , Zhao R, Wu R. An overview of the prediction of protein DNA-binding sites. Int J Mol Sci2015b;16:5194–215. 25756377 10.3390/ijms16035194 PMC4394471

[vbad184-B26] Tunyasuvunakool K , Adler J, Wu Z et al Highly accurate protein structure prediction for the human proteome. Nature 2021;596:590–6. 34293799 10.1038/s41586-021-03828-1 PMC8387240

[vbad184-B27] Uversky VN , Kurgan L. Overview update: computational prediction of intrinsic disorder in proteins. Curr Protoc 2023;3:e802. 37310199 10.1002/cpz1.802

[vbad184-B28] Varadi M , Anyango S, Deshpande M et al AlphaFold protein structure database: massively expanding the structural coverage of protein-sequence space with high-accuracy models. Nucleic Acids Res 2022;50:D439–44. 34791371 10.1093/nar/gkab1061 PMC8728224

[vbad184-B29] Varadi M , Vranken W, Guharoy M et al Computational approaches for inferring the functions of intrinsically disordered proteins. Front Mol Biosci 2015;2:45. 26301226 10.3389/fmolb.2015.00045 PMC4525029

[vbad184-B30] Wilson CJ , Choy WY, Karttunen M. AlphaFold2: a role for disordered protein/region prediction? Int J Mol Sci 2022;23:4591. 35562983 10.3390/ijms23094591 PMC9104326

[vbad184-B31] Wren JD. Bioinformatics programs are 31-fold over-represented among the highest impact scientific papers of the past two decades. Bioinformatics 2016;32:2686–91. 27153671 10.1093/bioinformatics/btw284

[vbad184-B32] Wren JD , Georgescu C, Giles CB et al Use it or lose it: citations predict the continued online availability of published bioinformatics resources. Nucleic Acids Res 2017;45:3627–33. 28334982 10.1093/nar/gkx182 PMC5397159

[vbad184-B33] Yan J , Friedrich S, Kurgan L. A comprehensive comparative review of sequence-based predictors of DNA- and RNA-binding residues. Brief Bioinform 2016;17:88–105. 25935161 10.1093/bib/bbv023

[vbad184-B34] Zhang H , Zhang T, Chen K et al Critical assessment of high-throughput standalone methods for secondary structure prediction. Brief Bioinform 2011;12:672–88. 21252072 10.1093/bib/bbq088

[vbad184-B35] Zhang Y , Bao W, Cao Y et al A survey on protein–DNA-binding sites in computational biology. Brief Funct Genomics 2022;21:357–75. 35652477 10.1093/bfgp/elac009

[vbad184-B36] Zhao B , Ghadermarzi S, Kurgan L. Comparative evaluation of AlphaFold2 and disorder predictors for prediction of intrinsic disorder, disorder content and fully disordered proteins. Comput Struct Biotechnol J 2023;21:3248–58. 10.1016/j.csbj.2023.06.001 PMC10782001 38213902

[vbad184-B37] Zhao B , Kurgan L. Surveying over 100 predictors of intrinsic disorder in proteins. Expert Rev Proteomics 2021;18:1019–29. 34894985 10.1080/14789450.2021.2018304

[vbad184-B38] Zhao B , Kurgan L. Deep learning in prediction of intrinsic disorder in proteins. Comput Struct Biotechnol J 2022;20:1286–94. 35356546 10.1016/j.csbj.2022.03.003 PMC8927795

[vbad184-B39] Zhao B , Kurgan L. Machine learning for intrinsic disorder prediction. In: Kurgan L (ed.), Machine Learning in Bioinformatics of Protein Sequences. Singapore: World Scientific Publishing, 2023, 205–36.

