# Supplementary material for: Availability of web servers significantly boosts citations rates of bioinformatics methods for protein function and disorder prediction
Source: Bioinform Adv. 2023 Dec 25;3(1):vbad184. doi: 10.1093/bioadv/vbad184 (PMC10749743; doi:10.1093/bioadv/vbad184)
Supplement: vbad184_Supplementary_Data [file vbad184_supplementary_data.pdf]

Supplement

Suppl. Table S1. Sequence-based predictors of functions annotated from structured complexes (protein-binding, peptide-binding, DNA-binding, and RNA-binding) that were published during the 10-year period between 2012 and 2021. Methods are sorted chronologically. "Mode of availability" column covers web server (WS), standalone code (SC), and description only with no standalone code and web server (no SC/WS). "URL" gives pages where a given method was available as of May 2023. Citations data were collected from Google Scholar in May 2023, where annual citation rate is computed as the total number of citations divided by the number of years since publication.

Method [reference]	Year published	Target of prediction	Mode of availability	URL (or specified as no longer working as of May 2023)	Total citations	Annual citation rate
NCBRPred [1]	2021	DNA/RNA-binding	WS+SC	http://bliulab.net/NCBRPred/	17	8.5
DNAgenie [2]	2021	DNA-binding	WS	http://biomine.cs.vcu.edu/servers/DNAgenie/	7	3.5
method by Zhang et al. [3]	2021	Protein-binding	SC	https://github.com/biolushuai/attention-based-CNNs-for-PPIs-prediction	4	2.0
PPISP-XGBoost [4]	2021	Protein-binding	SC	https://github.com/QUST-AIBBDRC/PPISP-XGBoost/	28	14.0
DELPHI [5]	2021	Protein-binding	WS+SC	http://www.csd.uwo.ca/~yli922/index.php	58	29.0
DeepPPISP-XGB [6]	2021	Protein-binding	SC	https://github.com/fatancy2580/DeepPPISPXGB-master	3	1.5
HANPPIS [7]	2021	Protein-binding	no SC/WS	no SC/WS	3	1.5
ProNA2020 [8]	2020	Protein/DNA/RNA-binding	WS+SC	http://www.predictprotein.org/	47	15.7
method by Shu et al. [9]	2020	Protein-binding	SC	https://github.com/Xiaoya-Deng/PPI-sites-prediction	33	11.0
DeepPPISP [10]	2020	Protein-binding	WS+SC	http://bioinformatics.csu.edu.cn/PPISP/	161	53.7
method by Wang et al. [11]	2020	Protein-binding	no SC/WS	no SC/WS	43	14.3
hybridNAP [12]	2019	Protein/DNA/RNA-binding	WS	http://biomine.cs.vcu.edu/servers/hybridNAP/	85	21.3
RNA-CNN [13]	2019	RNA-binding	no SC/WS	no SC/WS	2	0.5
DNApred [14]	2019	DNA-binding	WS	The website was not accessible as of May 2023	38	9.5
NucBind [15]	2019	DNA/RNA-binding	WS	The website was not accessible as of May 2023	44	11.0
SCRIBER [16]	2019	Protein-binding	WS	http://biomine.cs.vcu.edu/servers/SCRIBER/	81	20.3
DLPred [17]	2019	Protein-binding	WS+SC	The website was not accessible as of May 2023	62	15.5
EL-SMURF [18]	2019	Protein-binding	SC	https://github.com/QUST-AIBBDRC/EL-SMURF/	116	29.0
DRNApred [19]	2017	DNA/RNA-binding	WS	http://biomine.cs.vcu.edu/servers/DRNApred/	158	26.3
PredRBR [20]	2017	RNA-binding	SC	The website was not accessible as of May 2023	39	6.5
DORAEMON [21]	2017	RNA-binding	SC	https://github.com/ABCgrp/DORAEMON/	6	1.0
TargetDNA [22]	2016	DNA-binding	WS	The website was not accessible as of May 2023	61	8.7
FastRNABindR [23]	2016	RNA-binding	WS+SC	The website was not accessible as of May 2023	16	2.3
RNAProSite [24]	2016	RNA-binding	WS	The website was not accessible as of May 2023	27	3.9
SSWRF [25]	2016	Protein-binding	WS+SC	The website was not accessible as of May 2023	103	14.7
SPRINT [26]	2016	Peptide-binding	WS	The website was not accessible as of May 2023	87	12.4
iPPBS-Opt [27]	2016	Protein-binding	WS	http://www.jci-bioinfo.cn/iPPBS-Opt	170	24.3
PPIS [28]	2016	Protein-binding	SC	http://csbio.njust.edu.cn/bioinf/PPIS	42	6.0
SNBRFinder [29]	2015	DNA/RNA-binding	WS	The website was not accessible as of May 2023	27	3.4
CRF-PPI [30]	2015	Protein-binding	SC	The website was not accessible as of May 2023	48	6.0
method by Geng et al. [31]	2015	Protein-binding	no SC/WS	no SC/WS	38	4.8
aaRNA [32]	2014	RNA-binding	WS	http://sysimm.ifrec.osaka-u.ac.jp/aarna/	54	6.0
RNABindRPlus [33]	2014	RNA-binding	WS	The website was not accessible as of May 2023	95	10.6
method by Wang et al. [34]	2014	Protein-binding	no SC/WS	no SC/WS	72	8.0
SPRINGS [35]	2014	Protein-binding	SC	https://sites.google.com/site/predppis/	53	5.9
LORIS [36]	2014	Protein-binding	SC	The website was not accessible as of May 2023	76	8.4
DNABR [37]	2012	DNA-binding	WS	The website was not accessible as of May 2023	57	5.2
SRCpred [38]	2011	RNA-binding	WS	The website was not accessible as of May 2023	40	3.3
PredictRBP [39]	2011	RNA-binding	SC	The website was not accessible as of May 2023	39	3.3
Choi and Han method [40]	2011	RNA-binding	no SC/WS	no SC/WS	31	2.6
PRBR [41]	2011	RNA-binding	WS	The website was not accessible as of May 2023	73	6.1
SPOT-Seq-RNA [42]	2011	RNA-binding	WS	The website was not accessible as of May 2023	62	5.2
HomPPI [43]	2011	Protein-binding	WS	The website was not accessible as of May 2023	106	8.8

Suppl. Table S2. Sequence-based predictors of disorder functions (MoRFs, protein-binding IDRs, DNA-binding IDRs, RNA-binding IDRs, lipid-binding IDRs and linkers) that were published during the 10-year period between 2012 and 2021. Methods are sorted chronologically. “Mode of availability” column covers web server (WS), standalone code (SC), and description only with no standalone code and web server (no SC/WS). “URL” gives pages where a given method was available as of May 2023. Citations data were collected from Google Scholar in May 2023, where annual citation rate is computed as the total number of citations divided by the number of years since publication.

Method [reference]	Year published	Target of prediction	Mode of availability	URL (or specified as no longer working as of May 2023)	Total citations	Annual citation rate
DisoLipPred [44]	2021	Lipid-binding	WS	http://biomine.cs.vcu.edu/servers/DisoLipPred/	24	12.0
$MoRF_{CNN}$ [45]	2021	MoRFs	no SC/WS	no SC/WS	5	2.5
DeepDISObind [46]	2021	Protein/DNA/RNA-binding	WS+SC	https://www.csuligroup.com/DeepDISOBind/	23	11.5
MemDis [47]	2021	Lipid-binding	WS+SC	http://memdis.ttk.hu/	9	4.5
APOD [48]	2020	Linkers	WS	https://yanglab.nankai.edu.cn/APOD/	16	5.3
SPOT-MoRF [49]	2020	MoRFs	WS+SC	https://sparks-lab.org/server/spot-morf/	36	12.0
IDRBind [50]	2019	Protein-binding	WS	The website was not accessible as of May 2023	4	1.0
OPAL+ [51]	2019	MoRFs	WS+SC	http://www.alok-ai-lab.com/tools/opal_plus/	37	9.3
en_DCNNMoRF [52]	2019	MoRFs	WS	The website was not accessible as of May 2023	14	3.5
$MoRF_{MPM}$ [53]	2019	MoRFs	SC	https://github.com/HHJHgithub/MoRFs_MPM	8	2.0
MoRFPred_en [54]	2019	MoRFs	WS	The website was not accessible as of May 2023	8	2.0
$MoRF_{MLP}$ [55]	2019	MoRFs	no SC/WS	no SC/WS	9	2.3
Fang et al. [56]	2018	MoRFs	no SC/WS	no SC/WS	8	1.6
MoRFPred-plus [57]	2018	MoRFs	SC	https://github.com/roneshsharma/MoRFpred-plus/wiki/MoRFpred-plus	48	9.6
OPAL [58]	2018	MoRFs	WS+SC	http://www.alok-ai-lab.com/tools/opal/	61	12.2
ANCHOR2 [59]	2018	Protein-binding	WS+SC	http://iupred2a.elte.hu	545	109.0
DFLpred [60]	2016	Linkers	WS+SC	http://biomine.cs.vcu.edu/servers/DFLpred/	72	10.3
fMoRFpred [61]	2016	MoRFs	WS	http://biomine.cs.vcu.edu/servers/fMoRFpred/	136	19.4
MoRFCHiBi SYSTEM [62]	2016	MoRFs	WS+SC	https://gsponerlab.msl.ubc.ca/software/morf_chibi/	118	16.9
Predict-MoRFs [63]	2016	MoRFs	SC	https://github.com/roneshsharma/Predict-MoRFs	30	4.3
DisoRDPbind [64]	2015	Protein/DNA/RNA-binding	WS	http://biomine.cs.vcu.edu/servers/DisoRDPbind/	139	17.4
MoRFCHiBi [65]	2015	MoRFs	WS+SC	https://gsponerlab.msl.ubc.ca/software/morf_chibi/	83	10.4
MFSPSSMpred [66]	2013	MoRFs	WS+SC	The website was not accessible as of May 2023	60	6.0
MoRFpred [67]	2012	MoRFs	WS	http://biomine.cs.vcu.edu/servers/MoRFpred/	342	31.1

Suppl. Table S3. Sequence-based predictors of intrinsic disorder that were published during the 10-year period between 2012 and 2021. Methods are sorted chronologically. "Mode of availability" column covers web server (WS), standalone code (SC), and description only with no standalone code and web server (no SC/WS). "URL" gives pages where a given method was available as of May 2023. Citations data were collected from Google Scholar in May 2023, where annual citation rate is computed as the total number of citations divided by the number of years since publication.

**Method [reference]**	**Year published**	**Mode of availability**	**URL (or specified as no longer working as of May 2023)**	**Total citations**	**Annual citation rate**
IUPred3 [68]	2021	WS+SC	https://iupred3.elte.hu/	148	74.0
flDPnn [69]	2021	WS+SC	http://biomine.cs.vcu.edu/servers/flDPnn/	80	40.0
RFPR-IDP [70]	2021	WS	http://bliulab.net/RFPR-IDP/server	17	8.5
Metapredict [71]	2021	WS+SC	https://metapredict.net/	59	29.5
ODiNPred [72]	2020	WS	https://st-protein.chem.au.dk/odinpred	52	17.3
IDP-Seq2Seq [73]	2020	WS	http://bliulab.net/IDP-Seq2Seq/	73	24.3
rawMSA [74]	2019	SC	https://bitbucket.org/clami66/rawmsa/src/master/	58	14.5
SPOT-Disorder2 [75]	2019	WS+SC	https://sparks-lab.org/server/spot-disorder2/	94	23.5
Spark-IDPP [76]	2019	no SC/WS	no SC/WS	32	8.0
IDP-FSP [77]	2019	no SC/WS	no SC/WS	11	2.8
IUpred2A [78]	2018	WS+SC	https://iupred2a.elte.hu/	802	160.4
pyHCA [79]	2018	SC	https://github.com/T-B-F/pyHCA	5	1.0
SPOT-Disorder-Single [80]	2018	WS+SC	https://sparks-lab.org/server/spot-disorder-single/	60	12.0
Predictor by Zhao and Xue [81]	2018	no SC/WS	no SC/WS	12	2.4
IDP-CRF [82]	2018	no SC/WS	no SC/WS	22	4.4
MobiDB-lite [83]	2017	WS	http://mobidb.bio.unipd.it/	161	26.8
SPOT-Disorder [84]	2017	WS+SC	https://sparks-lab.org/server/spot-disorder/	259	43.2
AUCpred [85]	2016	SC	The website was not accessible as of May 2023	104	14.9
DISOPRED3 [86]	2015	WS+SC	http://bioinf.cs.ucl.ac.uk/psipred/	737	92.1
DisoMCS [87]	2015	WS	The website was not accessible as of May 2023	3	0.4
DeepCNF-D [88]	2015	SC	http://ttic.uchicago.edu/~wangsheng/DeepCNF_D_package_v1.00.tar.gz	76	9.5
DisMeta [89]	2014	WS	http://montelionelab.chem.rpi.edu/dismeta/	68	7.6
disCoP [90]	2014	WS	http://biomine.cs.vcu.edu/servers/disCoP/	161	17.9
DynaMine [91]	2014	WS+SC	https://bio2byte.be/dynamine/	152	16.9
MFDp2 [92]	2013	WS	http://biomine.cs.vcu.edu/servers/MFDp2/	95	9.5
DNdisorder [93]	2013	WS	The website was not accessible as of May 2023	103	10.3
x3Disorder [94]	2013	WS	The website was not accessible as of May 2023	9	0.9
Espritz [95]	2012	WS+SC	http://old.protein.bio.unipd.it/espritz/	446	40.5
GSmetadisorder [96]	2012	WS	http://iimcb.genesilico.pl/metadisorder/	359	32.6
SPINE-D [97]	2012	SC	The website was not accessible as of May 2023	184	16.7
DISOclust3 (IntFOLD) [98]	2011	WS+SC	http://www.reading.ac.uk/bioinf/DISOclust/	110	9.2
Cspritz [99]	2011	WS	The website was not accessible as of May 2023	101	8.4
IsUnstruct [100]	2011	WS+SC	http://bioinfo.protres.ru/IsUnstruct/	77	6.4

Suppl. Table S4. Predictive performance of the intrinsic disorder predictors that were published between 2012 and 2021 and which were evaluated using the CAID benchmark. Methods are sorted chronologically. The results were collected from refs [101, 102]. Citations data were collected from Google Scholar in May 2023, where annual citation rate is computed as the total number of citations divided by the number of years since publication.

Method [reference]	Has WS	Highly cited (annual citations≥40)	AUC	MCC
flDPnn [69]	yes	yes	0.814	0.327
RFPR-IDP [70]	yes	no	0.722	0.275
Metapredict [71]	yes	no	0.746	0.273
IDP-Seq2Seq [73]	yes	no	0.752	0.293
rawMSA [74]	no	no	0.780	0.325
SPOT-Disorder2 [75]	yes	no	0.760	0.343
IUpred2A [78]	yes	yes	0.741	0.278
pyHCA [79]	no	no	0.706	0.240
SPOT-Disorder-Single [80]	yes	no	0.757	0.278
MobiDB-lite [83]	yes	no	0.737	0.253
SPOT-Disorder [84]	yes	yes	0.744	0.311
AUCpred [85]	no	no	0.757	0.303
DISOPRED3 [86]	yes	yes	0.701	0.241
Espritz [95]	yes	yes	0.774	0.289
IsUnstruct [100]	yes	no	0.744	0.287

References

1. Zhang, J., Q. Chen, and B. Liu, *NCBRPred: predicting nucleic acid binding residues in proteins based on multilabel learning.* Brief Bioinform, 2021. **22**(5).
2. Zhang, J., et al., *DNAgenie: accurate prediction of DNA-type-specific binding residues in protein sequences.* Brief Bioinform, 2021. **22**(6).
3. Lu, S., et al. *Attention-based Convolutional Neural Networks for Protein-Protein Interaction Site Prediction*. in *2021 IEEE International Conference on Bioinformatics and Biomedicine (BIBM).* 2021.
4. Wang, X., et al., *Prediction of protein-protein interaction sites through eXtreme gradient boosting with kernel principal component analysis.* Computers in Biology and Medicine, 2021. **134**.
5. Li, Y., G.B. Golding, and L. Ilie, *DELPHI: accurate deep ensemble model for protein interaction sites prediction.* Bioinformatics, 2021. **37**(7): p. 896-904.
6. Wang, P., et al., *A Deep Learning and XGBoost-Based Method for Predicting Protein-Protein Interaction Sites.* Front Genet, 2021. **12**: p. 752732.
7. Tang, M.L., et al., *Prediction of Protein-Protein Interaction Sites Based on Stratified Attentional Mechanisms.* Frontiers in Genetics, 2021. **12**.
8. Qiu, J., et al., *ProNA2020 predicts protein-DNA, protein-RNA, and protein-protein binding proteins and residues from sequence.* J Mol Biol, 2020. **432**(7): p. 2428-2443.
9. Xie, Z.Y., X.Y. Deng, and K.X. Shu, *Prediction of Protein-Protein Interaction Sites Using Convolutional Neural Network and Improved Data Sets.* International Journal of Molecular Sciences, 2020. **21**(2).
10. Zeng, M., et al., *Protein-protein interaction site prediction through combining local and global features with deep neural networks.* Bioinformatics, 2020. **36**(4): p. 1114-1120.
11. Deng, A.J., et al., *Developing Computational Model to Predict Protein-Protein Interaction Sites Based on the XGBoost Algorithm.* International Journal of Molecular Sciences, 2020. **21**(7).
12. Zhang, J., Z. Ma, and L. Kurgan, *Comprehensive review and empirical analysis of hallmarks of DNA-, RNA- and protein-binding residues in protein chains.* Brief Bioinform, 2019. **20**(4): p. 1250-1268.

13. Ma, Y.C. and C.H. Yan, *A Concurrent Neural Network (CNN) Method for RNA-binding Site Prediction.* Proceedings of 2019 Ieee 8th Joint International Information Technology and Artificial Intelligence Conference (Itaic 2019), 2019: p. 567-570.
14. Zhu, Y.-H., et al., *DNAPred: accurate identification of DNA-binding sites from protein sequence by ensembled hyperplane-distance-based support vector machines.* Journal of chemical information and modeling, 2019. **59**(6): p. 3057-3071.
15. Su, H., et al., *Improving the prediction of protein-nucleic acids binding residues via multiple sequence profiles and the consensus of complementary methods.* Bioinformatics, 2019. **35**(6): p. 930-936.
16. Zhang, J. and L. Kurgan, *SCRIBER: accurate and partner type-specific prediction of protein-binding residues from proteins sequences.* Bioinformatics, 2019. **35**(14): p. i343-i353.
17. Zhang, B.Z., et al., *Sequence-based prediction of protein-protein interaction sites by simplified long short-term memory network.* Neurocomputing, 2019. **357**: p. 86-100.
18. Wang, X.Y., et al., *Protein-protein interaction sites prediction by ensemble random forests with synthetic minority oversampling technique.* Bioinformatics, 2019. **35**(14): p. 2395-2402.
19. Yan, J. and L. Kurgan, *DRNApred, fast sequence-based method that accurately predicts and discriminates DNA- and RNA-binding residues.* Nucleic Acids Res, 2017. **45**(10): p. e84.
20. Tang, Y., et al., *A boosting approach for prediction of protein-RNA binding residues.* BMC Bioinformatics, 2017. **18**(Suppl 13): p. 465.
21. Pai, P.P., T. Dash, and S. Mondal, *Sequence-based discrimination of protein-RNA interacting residues using a probabilistic approach.* Journal of Theoretical Biology, 2017. **418**: p. 77-83.
22. Hu, J., et al., *Predicting protein-DNA binding residues by weightedly combining sequence-based features and boosting multiple SVMs.* IEEE/ACM transactions on computational biology and bioinformatics, 2016. **14**(6): p. 1389-1398.
23. El-Manzalawy, Y., et al., *FastRNABindR: Fast and Accurate Prediction of Protein-RNA Interface Residues.* PLoS One, 2016. **11**(7): p. e0158445.
24. Sun, M., et al., *Accurate prediction of RNA-binding protein residues with two discriminative structural descriptors.* BMC Bioinformatics, 2016. **17**(1): p. 231.
25. Wei, Z.-S., et al., *Protein–protein interaction sites prediction by ensembling SVM and sample-weighted random forests.* Neurocomputing, 2016. **193**: p. 201-212.
26. Taherzadeh, G., et al., *Sequence-based prediction of protein–peptide binding sites using support vector machine.* Journal of computational chemistry, 2016.
27. Jia, J., et al., *iPPBS-Opt: a sequence-based ensemble classifier for identifying protein-protein binding sites by optimizing imbalanced training datasets.* Molecules, 2016. **21**(1): p. 95.
28. Liu, G.-H., H.-B. Shen, and D.-J. Yu, *Prediction of Protein–Protein Interaction Sites with Machine-Learning-Based Data-Cleaning and Post-Filtering Procedures.* The Journal of membrane biology, 2016. **249**(1-2): p. 141-153.
29. Yang, X., et al., *SNBRFinder: A Sequence-Based Hybrid Algorithm for Enhanced Prediction of Nucleic Acid-Binding Residues.* PLoS One, 2015. **10**(7): p. e0133260.
30. Wei, Z.-S., et al., *A Cascade Random Forests Algorithm for Predicting Protein-Protein Interaction Sites.* IEEE transactions on nanobioscience, 2015. **14**(7): p. 746-760.
31. Geng, H., et al., *Prediction of protein-protein interaction sites based on naive Bayes classifier.* Biochemistry research international, 2015. **2015**.
32. Li, S.L., et al., *Quantifying sequence and structural features of protein-RNA interactions.* Nucleic Acids Res, 2014. **42**(15): p. 10086-10098.
33. Walia, R.R., et al., *RNABindRPlus: A Predictor that Combines Machine Learning and Sequence Homology-Based Methods to Improve the Reliability of Predicted RNA-Binding Residues in Proteins.* Plos One, 2014. **9**(5).

34. Wang, D.D., R. Wang, and H. Yan, *Fast prediction of protein–protein interaction sites based on extreme learning machines.* Neurocomputing, 2014. **128**: p. 258-266.
35. Singh, G., et al., *SPRINGS: Prediction of Protein-Protein Interaction Sites Using Artificial Neural Networks.* J Proteomics Computational Biol, 2014. **1**.
36. Dhole, K., et al., *Sequence-based prediction of protein–protein interaction sites with L1-logreg classifier.* Journal of theoretical biology, 2014. **348**: p. 47-54.
37. Ma, X., et al., *Sequence-based prediction of DNA-binding residues in proteins with conservation and correlation information.* IEEE/ACM transactions on computational biology and bioinformatics, 2012. **9**(6): p. 1766-1775.
38. Fernandez, M., et al., *Prediction of dinucleotide-specific RNA-binding sites in proteins.* BMC Bioinformatics, 2011. **12 Suppl 13**: p. S5.
39. Wang, C.C., et al., *Identification of RNA-binding sites in proteins by integrating various sequence information.* Amino Acids, 2011. **40**(1): p. 239-248.
40. Choi, S. and K. Han, *Prediction of RNA-binding amino acids from protein and RNA sequences.* Bmc Bioinformatics, 2011. **12**.
41. Ma, X., et al., *Prediction of RNA-binding residues in proteins from primary sequence using an enriched random forest model with a novel hybrid feature.* Proteins-Structure Function and Bioinformatics, 2011. **79**(4): p. 1230-1239.
42. Zhao, H., Y. Yang, and Y. Zhou, *Highly accurate and high-resolution function prediction of RNA binding proteins by fold recognition and binding affinity prediction.* RNA Biol, 2011. **8**(6): p. 988-96.
43. Xue, L.C., D. Dobbs, and V. Honavar, *HomPPI: a class of sequence homology based protein-protein interface prediction methods.* BMC bioinformatics, 2011. **12**(1): p. 244.
44. Katuwawala, A., B. Zhao, and L. Kurgan, *DisoLipPred: accurate prediction of disordered lipid-binding residues in protein sequences with deep recurrent networks and transfer learning.* Bioinformatics, 2022. **38**(1): p. 115-124.
45. He, H., et al., *Prediction of MoRFs based on sequence properties and convolutional neural networks.* Biodata Mining, 2021. **14**(1).
46. Zhang, F.H., et al., *DeepDISOBind: accurate prediction of RNA-, DNA- and protein-binding intrinsically disordered residues with deep multi-task learning.* Briefings in Bioinformatics, 2021. **23**(1).
47. Dobson, L. and G.E. Tusnady, *MemDis: Predicting Disordered Regions in Transmembrane Proteins.* Int J Mol Sci, 2021. **22**(22).
48. Peng, Z., Q. Xing, and L. Kurgan, *APOD: accurate sequence-based predictor of disordered flexible linkers.* Bioinformatics, 2020. **36**(Supplement_2): p. i754-i761.
49. Hanson, J., et al., *Identifying molecular recognition features in intrinsically disordered regions of proteins by transfer learning.* Bioinformatics, 2020. **36**(4): p. 1107-1113.
50. Wong, E.T.C. and J. Gsponer, *Predicting Protein-Protein Interfaces that Bind Intrinsically Disordered Protein Regions.* Journal of Molecular Biology, 2019. **431**(17): p. 3157-3178.
51. Sharma, R., et al., *OPAL plus : Length-Specific MoRF Prediction in Intrinsically Disordered Protein Sequences.* Proteomics, 2019. **19**(6).
52. Fang, C., et al., *Identifying short disorder-to-order binding regions in disordered proteins with a deep convolutional neural network method.* Journal of Bioinformatics and Computational Biology, 2019. **17**(1).
53. He, H., J.X. Zhao, and G.L. Sun, *Computational prediction of MoRFs based on protein sequences and minimax probability machine.* Bmc Bioinformatics, 2019. **20**(1).
54. Fang, C., et al., *MoRFPred_en: Sequence-based prediction of MoRFs using an ensemble learning strategy.* Journal of Bioinformatics and Computational Biology, 2019. **17**(6).

55. He, H., J.X. Zhao, and G.L. Sun, *Prediction of MoRFs in Protein Sequences with MLPs Based on Sequence Properties and Evolution Information.* Entropy, 2019. **21**(7).
56. Fang, C., et al., *Identifying MoRFs in Disordered Proteins Using Enlarged Conserved Features.* Proceedings of 2018 6th International Conference on Bioinformatics and Computational Biology (Icbcb 2018), 2018: p. 50-54.
57. Sharma, R., et al., *MoRFPred-plus: Computational Identification of MoRFs in Protein Sequences using Physicochemical Properties and HMM profiles.* J Theor Biol, 2018. **437**: p. 9-16.
58. Sharma, R., et al., *OPAL: prediction of MoRF regions in intrinsically disordered protein sequences.* Bioinformatics, 2018. **34**(11): p. 1850-1858.
59. Meszaros, B., G. Erdos, and Z. Dosztanyi, *IUPred2A: context-dependent prediction of protein disorder as a function of redox state and protein binding.* Nucleic Acids Research, 2018. **46**(W1): p. W329-W337.
60. Meng, F. and L. Kurgan, *DFLpred: High-throughput prediction of disordered flexible linker regions in protein sequences.* Bioinformatics, 2016. **32**(12): p. i341-i350.
61. Yan, J., et al., *Molecular recognition features (MoRFs) in three domains of life.* Molecular Biosystems, 2016. **12**(3): p. 697-710.
62. Malhis, N., M. Jacobson, and J. Gsponer, *MoRFchibi SYSTEM: software tools for the identification of MoRFs in protein sequences.* Nucleic Acids Research, 2016. **44**(W1): p. W488-W493.
63. Sharma, R., et al., *Predicting MoRFs in protein sequences using HMM profiles.* Bmc Bioinformatics, 2016. **17**.
64. Peng, Z.L. and L. Kurgan, *High-throughput prediction of RNA, DNA and protein binding regions mediated by intrinsic disorder.* Nucleic Acids Research, 2015. **43**(18).
65. Malhis, N. and J. Gsponer, *Computational identification of MoRFs in protein sequences.* Bioinformatics, 2015. **31**(11): p. 1738-44.
66. Fang, C., et al., *MFSPSSMpred: identifying short disorder-to-order binding regions in disordered proteins based on contextual local evolutionary conservation.* Bmc Bioinformatics, 2013. **14**.
67. Disfani, F.M., et al., *MoRFpred, a computational tool for sequence-based prediction and characterization of short disorder-to-order transitioning binding regions in proteins.* Bioinformatics, 2012. **28**(12): p. i75-83.
68. Erdos, G., M. Pajkos, and Z. Dosztanyi, *IUPred3: prediction of protein disorder enhanced with unambiguous experimental annotation and visualization of evolutionary conservation.* Nucleic Acids Res, 2021. **49**(W1): p. W297-W303.
69. Hu, G., et al., *flDPnn: Accurate intrinsic disorder prediction with putative propensities of disorder functions.* Nature Communications, 2021. **12**(1): p. 4438.
70. Liu, Y., X. Wang, and B. Liu, *RFPR-IDP: reduce the false positive rates for intrinsically disordered protein and region prediction by incorporating both fully ordered proteins and disordered proteins.* Brief Bioinform, 2021. **22**(2): p. 2000-2011.
71. Emenecker, R.J., D. Griffith, and A.S. Holehouse, *Metapredict: a fast, accurate, and easy-to-use predictor of consensus disorder and structure.* Biophys J, 2021. **120**(20): p. 4312-4319.
72. Dass, R., F.A.A. Mulder, and J.T. Nielsen, *ODiNPred: comprehensive prediction of protein order and disorder.* Sci Rep, 2020. **10**(1): p. 14780.
73. Tang, Y.J., Y.H. Pang, and B. Liu, *IDP-Seq2Seq: identification of intrinsically disordered regions based on sequence to sequence learning.* Bioinformatics, 2021. **36**(21): p. 5177-5186.
74. Mirabello, C. and B. Wallner, *rawMSA: End-to-end Deep Learning using raw Multiple Sequence Alignments.* PLoS One, 2019. **14**(8): p. e0220182.
75. Hanson, J., et al., *SPOT-Disorder2: Improved Protein Intrinsic Disorder Prediction by Ensembled Deep Learning.* Genomics Proteomics Bioinformatics, 2019. **17**(6): p. 645-656.

76. Malysiak-Mrozek, B., T. Baron, and D. Mrozek, *Spark-IDPP: high-throughput and scalable prediction of intrinsically disordered protein regions with Spark clusters on the Cloud.* Cluster Computing-the Journal of Networks Software Tools and Applications, 2019. **22**(2): p. 487-508.
77. Liu, Y., et al., *Identification of Intrinsically Disordered Proteins and Regions by Length-Dependent Predictors Based on Conditional Random Fields.* Mol Ther Nucleic Acids, 2019. **17**: p. 396-404.
78. Meszaros, B., G. Erdos, and Z. Dosztanyi, *IUPred2A: context-dependent prediction of protein disorder as a function of redox state and protein binding.* Nucleic Acids Res, 2018. **46**(W1): p. W329-W337.
79. Bitard-Feildel, T. and I. Callebaut, *HCAtk and pyHCA: A Toolkit and Python API for the Hydrophobic Cluster Analysis of Protein Sequences*. 2018, bioRxiv.
80. Hanson, J., K. Paliwal, and Y. Zhou, *Accurate Single-Sequence Prediction of Protein Intrinsic Disorder by an Ensemble of Deep Recurrent and Convolutional Architectures.* J Chem Inf Model, 2018. **58**(11): p. 2369-2376.
81. Zhao, B. and B. Xue, *Decision-Tree Based Meta-Strategy Improved Accuracy of Disorder Prediction and Identified Novel Disordered Residues Inside Binding Motifs.* Int J Mol Sci, 2018. **19**(10).
82. Liu, Y.M., X.L. Wang, and B. Liu, *IDP-CRF: Intrinsically Disordered Protein/Region Identification Based on Conditional Random Fields.* International Journal of Molecular Sciences, 2018. **19**(9).
83. Necci, M., et al., *MobiDB-lite: fast and highly specific consensus prediction of intrinsic disorder in proteins.* Bioinformatics, 2017. **33**(9): p. 1402-1404.
84. Hanson, J., et al., *Improving protein disorder prediction by deep bidirectional long short-term memory recurrent neural networks.* Bioinformatics, 2017. **33**(5): p. 685-692.
85. Wang, S., J. Ma, and J. Xu, *AUCpreD: proteome-level protein disorder prediction by AUC-maximized deep convolutional neural fields.* Bioinformatics, 2016. **32**(17): p. i672-i679.
86. Jones, D.T. and D. Cozzetto, *DISOPRED3: precise disordered region predictions with annotated protein-binding activity.* Bioinformatics, 2015. **31**(6): p. 857-63.
87. Wang, Z., et al., *DisoMCS: Accurately Predicting Protein Intrinsically Disordered Regions Using a Multi-Class Conservative Score Approach.* PLoS One, 2015. **10**(6): p. e0128334.
88. Wang, S., et al., *DeepCNF-D: Predicting Protein Order/Disorder Regions by Weighted Deep Convolutional Neural Fields.* Int J Mol Sci, 2015. **16**(8): p. 17315-30.
89. Huang, Y.J., T.B. Acton, and G.T. Montelione, *DisMeta: a meta server for construct design and optimization.* Methods Mol Biol, 2014. **1091**: p. 3-16.
90. Fan, X. and L. Kurgan, *Accurate prediction of disorder in protein chains with a comprehensive and empirically designed consensus.* J Biomol Struct Dyn, 2014. **32**(3): p. 448-64.
91. Cilia, E., et al., *From protein sequence to dynamics and disorder with DynaMine.* Nat Commun, 2013. **4**: p. 2741.
92. Mizianty, M.J., Z. Peng, and L. Kurgan, *MFDp2: Accurate predictor of disorder in proteins by fusion of disorder probabilities, content and profiles.* Intrinsically Disord Proteins, 2013. **1**(1): p. e24428.
93. Eickholt, J. and J. Cheng, *DNdisorder: predicting protein disorder using boosting and deep networks.* BMC Bioinformatics, 2013. **14**: p. 88.
94. Becker, J., F. Maes, and L. Wehenkel, *On the encoding of proteins for disordered regions prediction.* PLoS One, 2013. **8**(12): p. e82252.
95. Walsh, I., et al., *ESpritz: accurate and fast prediction of protein disorder.* Bioinformatics, 2012. **28**(4): p. 503-9.
96. Kozlowski, L.P. and J.M. Bujnicki, *MetaDisorder: a meta-server for the prediction of intrinsic disorder in proteins.* BMC Bioinformatics, 2012. **13**: p. 111.
97. Zhang, T., et al., *SPINE-D: accurate prediction of short and long disordered regions by a single neural-network based method.* J Biomol Struct Dyn, 2012. **29**(4): p. 799-813.

98. Roche, D.B., et al., *The IntFOLD server: an integrated web resource for protein fold recognition, 3D model quality assessment, intrinsic disorder prediction, domain prediction and ligand binding site prediction.* Nucleic Acids Res, 2011. **39**(Web Server issue): p. W171-6.
99. Walsh, I., et al., *CSpritz: accurate prediction of protein disorder segments with annotation for homology, secondary structure and linear motifs.* Nucleic Acids Res, 2011. **39**(Web Server issue): p. W190-6.
100. Lobanov, M.Y. and O.V. Galzitskaya, *The Ising model for prediction of disordered residues from protein sequence alone.* Phys Biol, 2011. **8**(3): p. 035004.
101. Necci, M., et al., *Critical assessment of protein intrinsic disorder prediction.* Nat Methods, 2021. **18**(5): p. 472-481.
102. Zhao, B. and L. Kurgan, *Deep learning in prediction of intrinsic disorder in proteins.* Comput Struct Biotechnol J, 2022. **20**: p. 1286-1294.
